# Reviewer acknowledgement

**DOI:** 10.1186/1750-1326-9-9

**Published:** 2014-03-20

**Authors:** Guojun Bu, Huaxi Xu

**Affiliations:** 1 Department of Neuroscience, Mayo Clinic, Jacksonville, Florida, USA; 2 Sanford-Burnham Medical Research Institute, La Jolla, California, USA

## Abstract

**Contributing reviewers:**

The editors of *Molecular Neurodegeneration* would like to thank all the reviewers who have contributed to the journal in Volume 8 (2013).

## 

Samer Abdul-Hay

United States of America

Jeffrey Agar

United States of America

Alejandra Alonso

United States of America

Wim Annaert

Belgium

Ottavio Arancio

United States of America

Jesus Avila

Spain

Pascale Belenguer

France

Johannes Berger

Austria

Gal Bitan

United States of America

Kaj Blennow

Sweden

David Borchelt

United States of America

Ralf Braun

Germany

Marisa Brini

Italy

Saartje Burgmans

Netherlands

Jeffrey Burns

United States of America

Ruaidhri Carmody

United Kingdom

Anna Carrano

United States of America

Minerva Carrasquillo

United States of America

Eva Carro

Spain

Sally Chappell

United Kingdom

Frédéric Checler

France

Carlos Cruchaga

United States of America

Nadia D'Ambrosi

Italy

Pritam Das

United States of America

Peter Davies

United States of America

Ted Dawson

United States of America

Bart de Strooper

Belgium

Rahul Desikan

United States of America

Ilse Dewachter

Belgium

Dorothee Dormann

Germany

Piet Eikelenboom

Netherlands

Sophie Erhardt

Sweden

Steven Estus

United States of America

Anne Fagan

United States of America

Nick Fox

United Kingdom

Paul Fraser

Canada

John Fryer

United States of America

Yoshiaki Furukawa

Japan

Fen-Biao Gao

United States of America

Laura Gasparini

Italy

Tania Gendronn

United States of America

Tony Golde

United States of America

Sue Griffin

United States of America

Mariagrazia Grilli

Italy

Christian Hansen

Sweden

David Harris

United States of America

Takuya Hayashi

Japan

Emmanuel Hermans

Belgium

David Holtzman

United States of America

Jeroen Hoozemans

Netherlands

Bradley Hyman

United States of America

Haruhisa Inoue

Japan

Joanna Jankowsky

United States of America

Ashu Johri

United States of America

Jean-Pierre Julien

Canada

Takahisa Kanekiyo

United States of America

Satyabrata Kar

Canada

Eric Karran

United Kingdom

Jungsu Kim

United States of America

Deniz Kirik

Sweden

Radosveta Koldamova

United States of America

Dora M. Kovacs

United States of America

Anil Kumar

United States of America

Clotilde Lagier-Tourenne

United States of America

Gary Landreth

United States of America

Minglin Lang

United States of America

Weidong Le

United States of America

Tony Lefebvre

France

Efrat Levy

United States of America

Yueming Li

United States of America

Francesca-Fang Liao

United States of America

Gérard Lizard

France

Wenjie Luo

United States of America

Sanford Markey

United States of America

Larry Marsh

United States of America

Eduardo Martinez-Morillo

Canada

Eliezer Masliah

United States of America

Pamela McLean

United States of America

Christopher Medway

United States of America

Heather Melrose

United States of America

Michelle Mielke

United States of America

Kevin Morgan

United Kingdom

Sandra Mulder

Netherlands

Tomoyuki Nishizaki

Japan

Agneta Nordberg

Sweden

Karen O’Malley

United States of America

Harry Orr

United States of America

Thomas Pannicke

Germany

Robia Pautler

United States of America

Giuseppa Pennetta

United Kingdom

Richard Perrin

United States of America

Marina Pizzi

Italy

Erik Portelius

Sweden

Lawrence Rajendran

Switzerland

G. William Rebeck

United States of America

Ana Cristina Rego

Portugal

Marina Romero-Ramos

Denmark

Paul Rosenberg

United States of America

Terrone Rosenberry

United States of America

Bart Rutten

Netherlands

David Schaffer

United States of America

Michael Sendtner

Germany

Jie Shen

United States of America

Mitsuru Shinohara

United States of America

Ody Sibon

Netherlands

Sangram Sisodia

United States of America

Solomon Snyder

United States of America

Ioannis Sotiropoulos

Portugal

Nuno Sousa

Portugal

Wolfdieter Springer

United States of America

Harry Steinbusch

Netherlands

Grace Stutzmann

United States of America

Keizo Sugaya

Japan

Leon Tai

United States of America

Malu Tansey

United States of America

Gopal Thinakaran

United States of America

Taisuke Tomita

Japan

Ian Trounce

Australia

Brigita Urbanc

United States of America

Christine Vande Velde

Canada

Robert Vassar

United States of America

Marcel Verbeek

Netherlands

Aaron Voigt

Germany

Xin Wang

United States of America

Edwin Weeber

United States of America

Malin Wennström

Sweden

Patrick Weydt

Germany

Anthony White

Australia

Kristin Wildsmith

United States of America

Oliver Wirths

Germany

Benjamin Wolozin

United States of America

Zuoshang Xu

United States of America

X.William Yang

United States of America

Patrick Yu-Wai-Man

United Kingdom

Donald Zack

United States of America

Yun-wu Zhang

China

Hui Zheng

United States of America

Xiongwei Zhu

United States of America

Berislav Zlokovic

United States of America

